# Final height after gonadotropin-releasing hormone agonists with or without growth hormone in Korean girls with central precocious puberty and early puberty

**DOI:** 10.1186/1687-9856-2015-S1-P95

**Published:** 2015-04-28

**Authors:** Eun Byul Kwon, Young Suk Shim, Hae Sang Lee, Hwal Rim Jeong, Jin Soon Hwang

**Affiliations:** 1 Department of Pediatrics, Ajou University Hospital, School of Medicine, Ajou University, Suwon, Korea

## Aims

We investigated the final height (FH) in GnRHa treatment with or without GH in Korean girls with CPP or EP.

## Methods

The forty-one patients were divided retrospectively into three groups: group 1 subjects with CPP (n=20) received GnRHa. Group 2 with early puberty (n=12) received only GnRHa. Group 3 with early puberty (n=9) received combined treatment with GH and GnRHa.

## Results

The mean age at the start of treatment was 8.11 ± 0.70 years in group 1, 8.98 ± 0.38 years in group 2 and 9.46 ± 0.46 years in group 3, respectively. The mean predicted adult height (PAH) SDS at the start of treatment was -1.29 ± 1.16 in group 1, -1.14 ± 0.88 in group 2 and -1.87 ± 1.09 in group 3, respectively. Rate of growth during treatment with GnRHa combined with GH was higher significantly in group 3 (6.89 ± 1.45 cm) than in group 1 (5.27 ± 0.89 cm, p = 0.001) and in group 2 (5.64 ± 0.72 cm, p = 0.022). The mean FH SDS was -0.60 ± 0.88 in group 1, -0.40 ± 1.03 in group 2 and -0.92 ± 0.72, respectively and significantly higher than initial height prediction. For the girls received GnRHa alone, FH SDS was correlated significantly with TH SDS, PAH at the start of treatment, PAH at the discontinuation of treatment.

## Conclusion

After GnRHa treatment in girls with CPP or EP, FH is significantly higher than initial height prediction. GnRHa treatment combined with GH resulted in higher growth rate.

